# Improving Public Awareness of Climate Anxiety: A Medical Student Led Initiative

**DOI:** 10.1192/bjo.2024.300

**Published:** 2024-08-01

**Authors:** Athanasios Hassoulas, Abigail Finnie, Emily Shore

**Affiliations:** Cardiff University School of Medicine, Cardiff, United Kingdom

## Abstract

**Aims:**

Climate change, and the effects thereof, present challenges in all domains of life. Mental wellbeing is an often-overlooked area when considering the direct and indirect impact of climate uncertainty. Worrying about the outcome of current and future climate events and experiencing distress at the perceived lack of action taken by world leaders has given rise to reports of climate anxiety. Whilst not a diagnosable psychiatric illness, individuals experiencing climate anxiety report to experience excessive worry and fear that may impair activities of daily living. In addition, anxiety over the climate and environmental matters may exacerbate existing conditions such as generalised anxiety disorder (GAD).

**Methods:**

In an effort to raise public awareness of climate anxiety, a leaflet was designed by medical students for dissemination in General Practice surgeries, along with an interactive electronic version of the leaflet being made available for online dissemination. The World Health Organization's (WHO) guidance on health literacy in empowering communities and diverse audiences was adopted in the design of the leaflet. Key information was reported using interactive means that enabled the audience to engage with the content of the leaflet and to consider the impact of climate anxiety on mental wellbeing. A survey was embedded at the end of the leaflet, using a QR code, to collate feedback from the public and from clinicians on the usefulness and educational value of the leaflet.

**Results:**

The leaflet was shared with General Practitioners affiliated with the School of Medicine at Cardiff University, to disseminate at their surgeries, and was promoted by online and social media channels affiliated with the School of Medicine. Members of the public reported that the leaflet highlighted the importance of mental health considerations in relation to the climate crisis and provided a good overview of climate anxiety. Clinicians also reported the overall usefulness of the leaflet as a resource of information on climate anxiety.

**Conclusion:**

Climate anxiety is a relatively new phenomenon that most people are not familiar with or know little about. Raising public awareness of the impact the climate crisis might have on mental wellbeing is crucial. Of equal importance is improving clinical awareness of climate anxiety as a risk or perpetuating factor of existing anxiety and/or mood disorders, such as GAD.

